# Multi-omics sequencing revealed endostar combined with cisplatin treated non small cell lung cancer via anti-angiogenesis

**DOI:** 10.1186/s12885-023-11665-w

**Published:** 2024-02-08

**Authors:** Yufei Wang, Hong Ren

**Affiliations:** 1 Department of Thoracic Surgery, The First Affiliated Hospital of Xi’an Jiaotong University, No. 277 West Yanta Road, 710061 Shanxi, Xi’an, Shanxi P.R. China; 2 Department of Thoracic Surgery, The Affiliated Hospital of Inner Mongolia Medical University, 010050 Hohhot, Inner Mongolia P.R. China

**Keywords:** Non-small cell Lung cancer, Endostar, Combination therapy, m6A sequencing, Tumor angiogenesis, Cisplatin

## Abstract

**Background:**

Endostar, an anti-angiogenic drug, has been approved for treating non-small cell lung cancer (NSCLC). At present, endostar combined with radiotherapy or chemotherapy has achieved ideal results in the treatment of some tumors, but there is a lack of application and study in NSCLC. This study investigated the therapeutic effect and potential mechanism of endostar combined with cisplatin (EC) in NSCLC.

**Methods:**

HE staining, TUNEL staining, immunofluorescence, colony formation ability, and cell migration ability were used to evaluate the anti-tumor activity of EC. The expressions of FMOD, VEGF, FGF-2, and PDGF-B were detected by western blotting and qPCR. The target of combination therapy was analyzed by m6A sequencing and RNA sequencing. METTL3 knockdown and overexpressed A549 cells were constructed and co-cultured with HUVECs to further evaluate the effect of METLL3 on combination therapy.

**Results:**

Combination therapy significantly reduced the colony formation and migration ability of NSCLC cells, induced cell apoptosis, and inhibited the tube formation ability of HUVECs. The results of m6A sequencing and RNA sequencing showed that the EC could down-regulate the expression level of FMOD in tumor tissues, which might be related to the reduction of its m6A methylation modification regulatory enzyme METTL3. Restricting FMOD expression could reduce the expression of FGF2, TGF-β1, VEGF and PDGF-B. Moreover, overexpression of METTLE almost abolished the anti-tumor effect of EC and promoted angiogenesis.

**Conclusions:**

Endostar combined with cisplatin might exert anti-tumor effects by down-regulating the expression of METTL3 and FMOD.

**Supplementary Information:**

The online version contains supplementary material available at 10.1186/s12885-023-11665-w.

## Background

Lung cancer is one of the most common types of cancer and the leading cause of cancer-related death worldwide, with approximately 1.8 million patients dying from lung cancer according to Global Cancer Statistics 2020 [1]. Lung cancer is divided into small cell lung cancer (SCLC) and non small cell lung cancer (NSCLC). NSCLC accounts for over 80% of all lung cancer diagnoses [2]. The average 5-year survival rate for patients with NSCLC varies by stage of cancer, with survival rates ranging from 63% for stage I to 7% for stage IV [3]. Although early lung cancer is resectable for most patients, approximately 70% are diagnosed with advanced-stage lung cancer [4]. Therefore, accurate diagnosis and more effective treatment are important ways to improve the survival outcome of patients with NSCLC.

Treatment strategies for advanced NSCLC have advanced significantly in recent decades. Immunotherapy, specifically the development of antibodies that inhibit the CTLA-4 and PD-1/PD-L1 pathways, has improved survival outcomes in patients with advanced or metastatic NSCLC [5]. However, the primary resistance to immunotherapy means that only some patients benefit from immune checkpoint inhibitor therapy [6]. Therefore, the treatment plan of NSCLC still needs to be innovated continuously. Judah Folkman proposed for the first time the new concept that tumor growth depends on the angiogenesis process [7], which provided the theoretical basis for tumor anti-angiogenesis therapy. Fibromodulin (FMOD), belonging to the family of small leucine-rich proteoglycans, plays a role in the regulation of tumor angiogenesis [8]. Mondal et al. demonstrated that FMOD was aberrantly highly expressed in human glioblastoma tissues and enhances the migration of glioma cells by regulating actin cytoskeleton remodeling [9]. Further studies indicated that the demethylation level of FMOD promoter might be related to its expression level in tumor cells and its role in inducing angiogenesis [10]. These evidences suggested that FMOD might represent a promising target for anti-angiogenic therapeutic strategies. However, FMOD has not been adequately studied in NSCLC.

Endostar is a recombinant human endostatin independently developed in China and approved by the National Medical Products Administration for treating NSCLC in 2005 [11]. As an essential anti-angiogenic agent, endostar exerts anti-tumor angiogenic effects by blocking vascular endothelial growth factor (VEGF)-induced endothelial cell KDR/Flk-1 tyrosine phosphorylation [12]. Currently, endostar has been reported to have achieved good efficacy in the treatment of a variety of tumors, including lung cancer [13], nasopharyngeal carcinoma [14], and ovarian cancer [15]. However, treatment with anti-angiogenic drugs alone cannot cure the tumor [16]. A combination treatment regimen based on endostar may be a better option [17]. Cisplatin is one of the most effective oncologic chemotherapeutic agents widely used in clinical practice [18]. Cisplatin has been studied in many clinical trials as a stand-alone or combination therapy [19, 20]. It has been suggested that endostar could relieve pressure on tumor vessels, leading to increased vascular perfusion, thereby facilitating cisplatin transport and enhancing the effect of chemotherapy [21, 22]. Therefore, cisplatin combined with endostar is considered as an effective treatment option for tumors [23]. However, there are fewer studies on whether endostar in combination with cisplatin could treat NSCLC. In this study, we investigated the anti-tumor effect of endostar combined with cisplatin on NSCLC cells and the anti- angiogenesis in mice.

N6-Methyladenosine (m6A), the most abundant mRNA modification in eukaryotes, reversibly regulates RNA splicing and stability as well as translation of key oncogenes and is therefore closely associated with tumor development [24]. Emerging evidence that m6A modification is involved in tumor drug resistance. For example, methyltransferase-like 3 (METTL3) is defined as a key methylation enzyme as an m6A “writer”, and increased METTL3 expression was detected in osteosarcoma cells showing doxorubicin resistance [25]. Therefore, a comprehensive analysis of the m6A map is helpful in understanding the mechanism of endostar combined with cisplatin in the treatment of NSCLC.

In this study, we investigated the synergistic effect of endostar combined with cisplatin in the treatment of NSCLC and established the expression profile of M6A modification to explore the potential regulatory mechanism of M6A modification on combination therapy, which provides a reference for future clinical treatment.

## Materials and methods

### Nude mouse Tumor xenograft model and drug intervention

In this study, 8-week-old male BALB/c nude mice (Beijing Huafukang Biotechnology Co., LTD., License No.: SCXK (Beijing) 2019-0008) were used to construct a tumor xenograft model. All mice were fed adaptive for one week before the experiment. Mice were fed in cages in SPF animal houses with 5 mice per box on laminar flow racks with good ventilation, no noise, room temperature of 20–25℃, relative humidity of 40–70%, alternating light (8 am-8 pm) 12 h a day, and free drinking and eating. The animal experiment was approved by the Ethics Committee.

The tumor nodules of human transplantable lung adenocarcinoma A549 cells, which had grown well subcutaneously in nude mice, were obtained by cell culture in vitro. The tumor cell suspension was prepared by aseptic operation and inoculated into the subcutaneously in the armpit of nude mice with puncture needle, one piece/mouse (about 2 mm^3^). All tumor bearing mice were randomly divided into three groups: model group, endostar group, and endostar combined with cisplatin (EC) group. Five mice in each group. In the experimental group, pericutaneous administration began on the 15th day after tumor inoculation, once every 6 days, three times in common. Both cisplatin and endostar were administered by peritumoral injection at a dose of 3 mg/kg, and the dose of combination therapy (EC) group was 6 mg/kg (cisplatin + endostar = 3 mg/kg + 3 mg/kg). These therapeutic doses refer to previous studies that have proven effective in the treatment of cancer mice [26]. The model group was treated with 0.9% sterile sodium chloride solution 5 mL/kg for 3 times. At the end of the last dose (on the 18th day), mice were sacrificed by cervical dislocation after 1% isoflurane inhalation anesthesia, tumor tissue was then mice were euthanized, tumor tissue was stripped and weighed to calculate the difference among groups.

### Cell and culture conditions

Human lung cancer cell A549 was provided by the BeNa Culture Collection (BNCC337696, Henan province industrial microbial strains engineering technology research center, China). A549 cells were treated with RPMI 1640 medium containing 10% FBS and 1% penicillin-streptomycin. Human umbilical vein endothelial cells (HUVECs) were purchased from ATCC and cultured in special culture medium (Ham’s F-12 K + 0.1 mg/mL Heparin + 0.03-0.05 mg/mL ECGs+10% FBS+1% P/S, Procell Life Science&Technology Co.,Ltd., China). All cells were cultured under 5% CO_2_ and 37℃ saturated humidity.

### Hematoxylin-eosin (HE) staining

All tumor samples of mice were collected, then washed with pre-cooled PBS and soaked in 4% polyformaldehyde for 48 h. Tissue samples were routinely paraffin-embedded and sectioned using a paraffin slicer at a thickness of 5 μm. After staining, the sections were dehydrated with gradient ethanol and transparent with xylene, and sealed in neutral glue. The slices were observed under the microscope.

### Immunohistochemistry

Antigen repair was performed after dewaxing and hydration of paraffin sections. Paraffin sections were dewaxed and hydrated for antigen repair. Briefly, the sections were boiled in sodium citrate buffer solution in a microwave oven for 3 min, cooled to room temperature, and then boiled again. The sections were immersed in 3% H2O2 to eliminate endogenous peroxidase activity. Sections were incubated in 5% bovine serum albumin (A1128Gentihold, Beijing, China) for 20 min to block nonspecific binding, and incubated with primary antibody (1:200) overnight at 4℃, followed by secondary antibody (1:500) at 37℃ for 60 min. Then, sections were stained with DAB and restained with hematoxylin, fractionated with 1% hydrochloric acid alcohol, and returned to blue with ammonia; then, the sections were dehydrated with gradient alcohol, transparent with xylene, and sealed with neutral gum, and observed by microscope.

### qPCR

RNA was extracted from tumor tissues or cells on ice according to the instructions of the total RNA extraction kit (TRIzol, Invitrogen Life Technologies, New York), and the extracted RNA was dissolved in RNase ⁃free ddH2O, gently blown and mixed, and then detected by spectrophotometer. The RNA was reverse transcribed into cDNA and the expression of FMOD, VEGF, TGF-β1, platelet-derived growth factor subunit B (PDGF-B), fibroblast growth factor 2 (FGF-2) and METTL3 was detected by qPCR with the primer sequences shown in Table 1. GAPDH was used as an internal reference. Quantitative calculations were performed by the 2^−∆∆CT^ method. The experiment was repeated three times with 5 samples in each group.

**Table 1:** The primer sequence for qRT-PCR

Gene	Product Size (bp)	Primer Pair	Primer Sequence (5’-3’)
FMOD	210	Forward	5’-CAGACTTGCACACTCTCCGT-3’
		Reverse	5’- AGGGGTCGTAGTAGGTGGAC-3’
VEGF	175	Forward	5’-GTCCGATTGAGACCCTGGTG-3’
		Reverse	5’-ATCCGCATGATCTGCATGGT-3’
FGF-2	153	Forward	5’-GGCTGCTGGCTTCTAAGTGT-3’
		Reverse	5’-GTCCCGTTTTGGATCCGAGT-3’
PDGF-B	215	Forward	5’-TATGGAAACGTCTGCGGGAA-3’
		Reverse	5’-TCAGCCCCATCTTCATCTACG-3’
METTL3	275	Forward	5’-CCGTAGTGATAGTCCCGTGC-3’
		Reverse	5’-TGGCGTAGAGATGGCAAGAC-3’
GAPDH	200	Forward	5’-ACAGTCAGCCGCATCTTCTT-3’
		Reverse	5’-GACAAGCTTCCCGTTCTCAG-3’

### Western blotting

The lysate RIPA (adding protease inhibitor before using RIPA, Solarbio, R0010, and A8260) was added to 30 mg tumor tissue, homogenized on ice, and continued to lysate for 30 min. After centrifugation at 12,000 rpm/min for 15 min, the supernatant was taken to obtain protein samples. The protein concentration was detected by BCA method according to the manufacturer’s instructions (Solarbio, PC0020). Protein samples were added into 5 × Loading Buffer, mixed, and boiled at 100 ℃ for 10 min. Samples were added into 10% SDS polyacrylamide gel successively, then subjected to 80 V constant pressure electrophoresis and 100 V constant pressure membrane transfer. The target bands were selected and sealed with 5% BSA. The PVDF membrane was washed for 3 times with 1×PBST, 5 min each time. Then the corresponding primary antibody (FMOD antibody, 1: 1000; VEGF antibody, 1: 1000; TGF-β1 antibody, 1: 2000; FGF-2 antibody, 1: 1000; GAPDH antibody, 1: 1000; These antibodies were purchased from Proteintech Group, Inc, Wuhan, China. PDGF-B, 1:2000, Abcam) was added and kept in a shaker at 4 ℃ overnight. After washing off the primary antibody, the secondary antibody (1: 10,000, Proteintech Group, Inc) was added and incubated at room temperature for 2 h. The gray values of the target bands were analyzed by gel imaging analyzer. The experiment was repeated independently with three biological replications.

### TUNEL staining analysis

Apoptosis analysis was performed by TUNEL staining using an in-situ apoptosis detection kit (Beijing Sigma Biotechnology Co., Ltd.) according to the manufacturer’s instructions. Blue fluorescence shows the total distribution of tumor cells, and green fluorescence indicates the number of apoptotic tumor cells. The mean fluorescence intensity was calculated for each group and the differences between groups were compared. The experiment was repeated three times.

### Adenovirus Infection and transfection

In this study, three siRNA sequences targeting METTLE gene and one METTLE overexpression vector were constructed. Briefly, the expression vectors carrying pAdTrack-CMV-METTL3-siRNA, pAdTrack-CMV-NC-siRNA, pAdTrack-CMV-METTL3, and pAdTrack-CMV-pCDNA3.1 sequences were transfected into A549 with Lipo2000 (Invitrogen) for adenoviral packaging. Then the adenovirus was collected and concentrated by ultra-high speed centrifugation and dialysis. Finally, A549 cells were transfected using adenovirus. Stable expression cell systems were screened by complete medium containing puromycin. Total RNA was extracted with TRIzol reagent and validated by qPCR and western blotting for effective knockdown and overexpression. The constructed cell lines were also used for mouse tumor xenograft model experiments.

### Colony formation test

The cells in the logarithmic growth stage were digested with trypsin and counted, then the cell density of each group was adjusted to 300 cells /ml. 300 cells were suspended in the 1640 medium containing 10% FBS, and the cell plate was gently shaken to make the cells evenly dispersed. The cells were incubated in a cell culture incubator at 37 ℃ with a concentration of 5% CO2. After cell apposition, the cells were treated with drug-containing media for 48 h (endostar 200 µg/mL + cisplatin 2 µg/mL, the same drug doses were used to treat A549 cells in the following experiments). The cells were then incubated in drug-free medium for 10 days. The medium was changed promptly according to the change in pH. When visible cell clonal clusters appeared in the cell culture plate, the cell culture was terminated and washed 3 times with 2 mL PBS. After natural drying, the cells were fixed with paraformaldehyde for 15 min; after the paraformaldehyde was discarded and dried, the cells were stained with the crystal purple solution for 10 min, rinsed slowly with distilled water, and dried naturally at room temperature. Colony formation was observed. The number of cell colonies in each group was statistically recorded.

### Cell apoptosis was analyzed by flow cytometry

Cells were inoculated into 6-well plates with a density of 2 × 10^5^ cells/well, and when the cell density approached 70%, the corresponding drugs were added and treated for 48 h. The operation of the apoptosis detection kit was carried out according to the manufacturer’s instructions. The cells were digested with trypsin, washed with 2 mL PBS, centrifuged at 1000 rpm/min for 5 min, and the supernatant was discarded. The cells were re-suspended with FCM staining buffer and added into the flow sample tubes at 100 µL per tube. Then 195 µL binding solution was added into each tube, and 10 µL FITC and 10 µL PI were added. The tubes were incubated at 37℃ for 30 min, away from light, and detected by flow cytometry.

### Cell migration capacity assay

The wound healing assay was used to evaluate cell migration ability. Cells were inoculated in 6-well plate with a density of 4 × 10^5^ cells/well, and when fusion was close to 90%, cell monolayers were manually scratched with a 200 µL pipette tip. Then washed off the scraped cells with PBS and continue to culture for 48 h using drug-containing media. Wound closure was observed with an inverted microscope immediately after scratching and continued to be cultured for 24 and 48 h, respectively. The migration rate was quantified based on distance. Migration rate was calculated as follows: Migration rate = migration distance/ original distance. Repeat three times for each time point.

### Tube formation capacity assay

Matrigel (Corning, Tewksbury, MA, USA) was added to 24-well plate and incubated in a cell incubator for 30 min. Five groups of cells, A549, A549-si-METTL3, A549-overexpressing-METTL3, A549 + EC treatment, and A549-overexpressing-METTL3 + EC treatment, were co-cultured with HUVECs for 48 h. Then, HUVECs in each group were collected separately. The cells were added to the pretreated 24-well plate with a density of 8 × 10^4^ cells/well, and the tubular structure was observed after continuing the culture for 4 h. Three fields of view were randomly selected for each group under the microscope to count the number of tubes.

### MTT assay

Cell viability was assessed by MTT assay. Transfected and non-transfected A549 cells were mixed with HUVECs in equal amounts to form cell suspensions, inoculated at 5,000 cells per well on 96-well plates, and then treated with or without EC for 24 h. After incubation with MTT for 3 h, the absorbance was measured at 570 nm using an emicroplate reader. Cell viability = OD treated / OD control × 100%.

### RNA sequencing and m6A sequencing

Total RNA was extracted from mouse tumor tissue using TRIzol reagent according to the instructions. RNA purity and quantification were determined using a NanoDrop s2000 spectrophotometer (Thermo Scientific, USA). RNA integrity was assessed using Agilent 2100 Bioanalyzer (Agilent Technologies, Santa Clara, CA, USA). The transcriptome library was constructed according to the instructions using the VAHTS Universal V6 RNA-seq Library Prep kit. Transcriptome sequencing and m6A sequencing analysis were conducted by Shanghai OE Biotech. Co., Ltd. Briftly, m6A sequencing was performed by enriching 50 µg of total RNA for mRNA using Oligo-dT magnetic beads. The mRNA was then fragmented to a length of approximately 150 nt. The fragmented RNA was divided into two parts. One part was added to the immunomagnetic beads with pre-mixed m6A antibody to enrich for mRNA fragments containing m6A methylation. The other part was used as a control to construct a conventional transcriptome sequencing library directly. The m6A antibody immunomagnetic beads were enriched and mRNA fragments with m6A were recovered to construct conventional sequencing libraries according to the library building process of the transcriptome. The two sequencing libraries, the m6A-sequencing library and the RNA-sequencing library, were sequenced in high throughput on Illumina NovaSeq 6000 sequencing platform and PE150 sequencing mode, respectively.

The expression of protein-coding genes was calculated using FPKM method [27], using the following formula:

$$\text{F}\text{P}\text{K}\text{M}={10}^{9}\times \frac{\text{C}}{\text{N}\text{L}}$$

In this formula, C = $\text{t}\text{h}\text{e} \text{n}\text{u}\text{m}\text{b}\text{e}\text{r} \text{o}\text{f} \text{r}\text{e}\text{a}\text{d}\text{s} \text{u}\text{n}\text{i}\text{q}\text{u}\text{e}\text{l}\text{y} \text{a}\text{l}\text{i}\text{g}\text{n}\text{e}\text{d} \text{t}\text{o}$Gene X, N = the total number of reads uniquely aligned to all genes, L = the number of bases in gene A.

The DESeq2 software was used to normalize the counts of gene, and differentially expressed genes between EC group and model group (DEGs) were identified with |exp_log2FoldChange| ≥ 1, p < 0.05. The DEGs were analyzed for GO and KEGG functional enrichment using the R software (version 3.5.2) clusterprofile package [28–30]. The top 10 GO terms and pathways were selected for display based on p-values and enrichment levels.

### Statistical analysis of data

All data were analyzed using SPSS software (version 15.0.1). Student’s t-test was used to determine the statistical significance between groups. In all experiments, data from more than two groups were analyzed using one way ANOVA. Statistical significance: p *<* 0.05. All data were given as mean ± standard deviation (SD) and plotted with GraphPad Prism 8.0.

## Results

### Anti-tumor effect of endostar combined with cisplatin

In order to investigate the anti-tumor effect of endostar combined with cisplatin (EC) in NSCLC cells, MTT, colony formation, wound healing and flow cytometry assays were performed. As shown in Fig. S1A-D, compared to the control group, endostar or cisplatin treatment notably suppressed A549 cell viability, proliferation, migration and induced A549 cell apoptosis. As expected, compared to the endostar or cisplatin single treatment group, EC treatment further reduced A549 cell viability, proliferation, migration and induced A549 cell apoptosis (Fig. S1A-D). These data showed that EC could suppress NSCLC cell proliferation and migration in vitro.

**Fig. 1:**
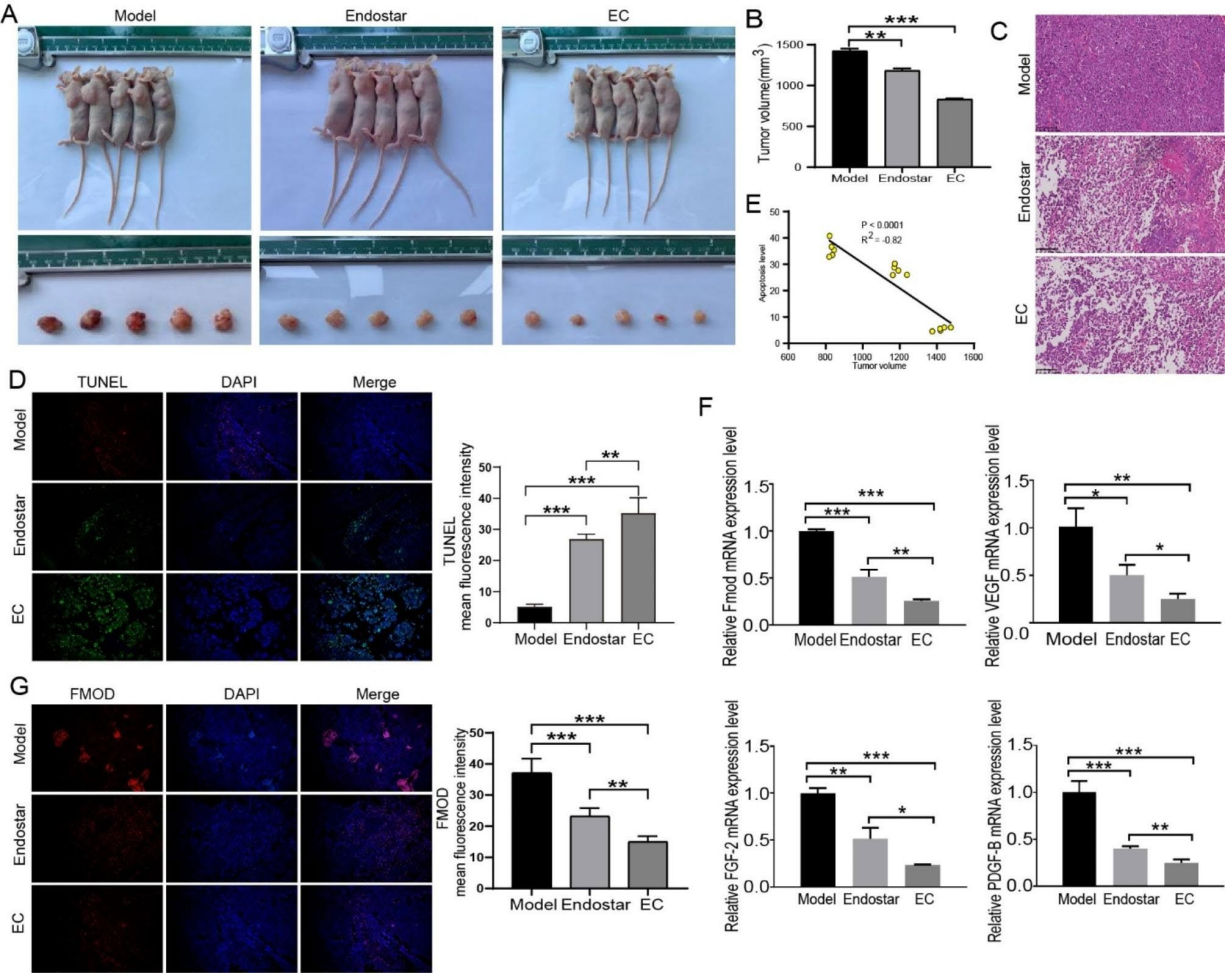
**Anti-tumor effect of endostar combined with cisplatin** in vivo. (**A**) Observation of tumor formation and size of xenograft tumor tissues in each group of nude mice (n = 5). (**B**) Tumor volume of mice on the 18th day (n = 5). (**C**) HE staining. Endostar combined with cisplatin could induce apoptosis of tumor cells. (**D**) TUNEL staining, blue fluorescence showed the total distribution of tumor cells, and green fluorescence indicated the number of apoptotic tumor cells. (**E**) Correlation analysis between tumor volume and apoptosis rate. (**F**) The expression of FMOD, VEGF, FGF-2 and PDGF-B was detected by qPCR (n = 5, mean ± SD). (**G**) The expression level of FMOD was detected by immunofluorescence. * p < 0.05, ** p < 0.01, *** p < 0.001, compared to the corresponding group

Next, we constructed a mouse tumor xenograft model for NSCLC to further explore the anti-tumor effect of EC in vivo. The results showed that the tumors of the mice were significantly inhibited, and the tumor growth rate was significantly lower than that of the model group after the combination therapy (Fig. 1A-B). HE staining showed that the tumor cells in the model group had large and darkly stained nuclei without prominent necrotic areas. In contrast, the EC group showed the highest level of apoptosis, the highest number of cell crumpling and larger necrotic areas. Thus we further confirmed that cisplatin combined with endostar treatment could significantly induce apoptosis and inhibit tumor proliferation (Fig. 1C). TUNEL staining further showed that the level of apoptosis was significantly increased in the EC group compared with endostar group (Fig. 1D). In this study, the mean fluorescence intensity of TUNEL was used to represent the level of cell apoptosis. The correlation between apoptosis level and tumor volume after treatment (on the 18th day after drug intervention) was analyzed, as shown in Fig. 1E. There was a significant negative correlation between apoptosis level and tumor volume, with R^2^ = -0.86. This indicated that the higher the level of apoptosis, the smaller the tumor volume.

Tumorigenesis, progression, and metastasis are closely related to angiogenesis [31]. Angiogenesis is associated with bioactive substances such as VEGF, FGF-2, and PDGFs [32]. Therefore, we further detected the expression levels of angiogenesis-related factors VEGF, FGF-2, FMOD, and PDGF-B in tumor tissues by qPCR. The results showed that the expression levels of FMOD, VEGF, FGF-2, and PDGF-B were decreased by endostar and further decreased after combination therapy (p < 0.05 or p < 0.01) (Fig. 1F). In addition, immunofluorescence results showed that compared with the model group, the expression level of FMOD was significantly decreased in the endostar group, and further decreased after combination therapy (Fig. 1G).

### Transcriptome analysis reveals FMOD as an important effector gene

**Fig. 2:**
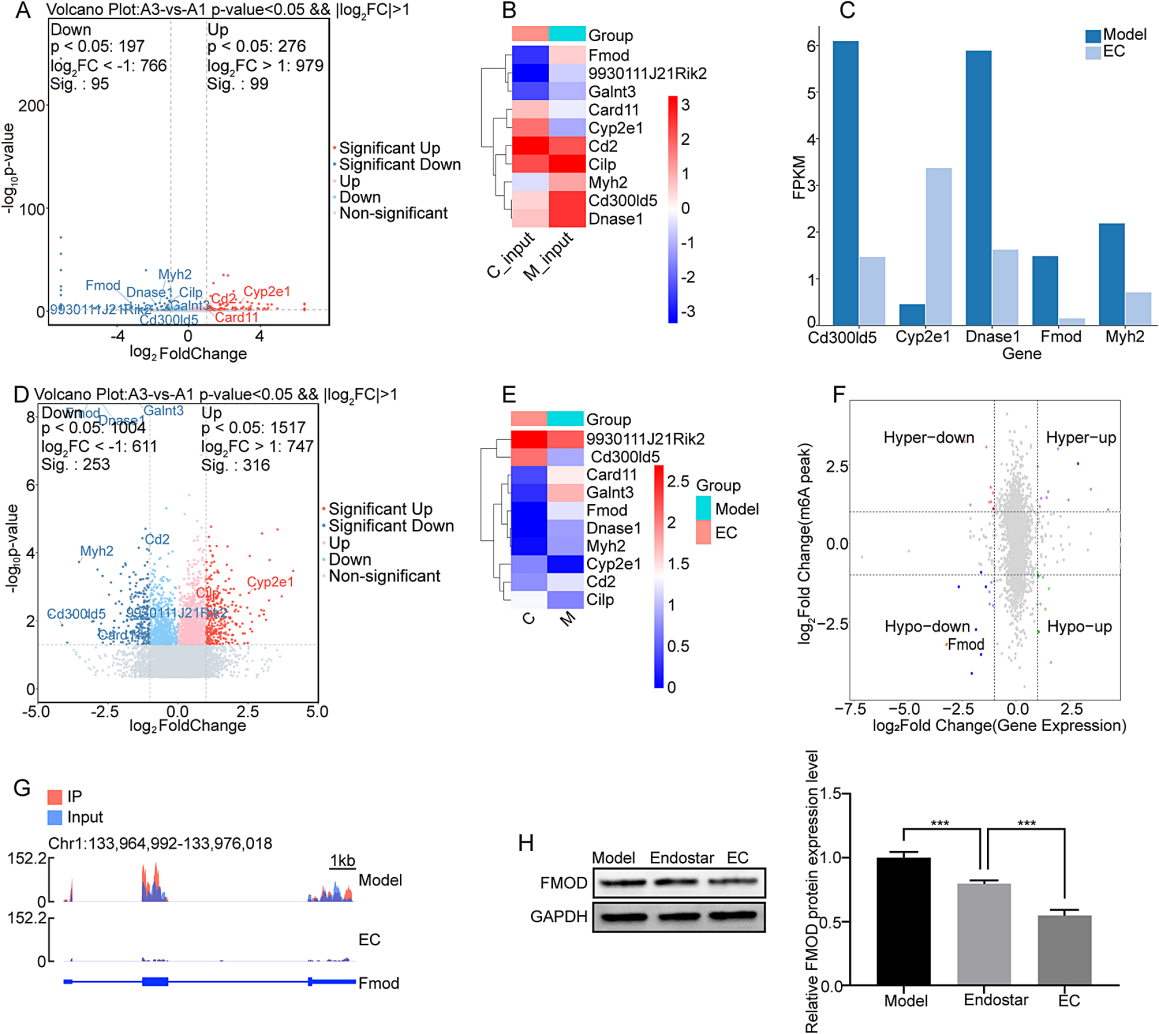
**High throughput sequencing analysis.** (**A**) Volcanic maps of DEGs between the model and the combined treatment group. (**B**) Heat maps of 10 DEGs. (**C**) Histogram of FPKM values. (**D**) Volcanic maps of differential m6A methylation modification between the model and the EC group. (**E**) m6A methylation modification heat maps of 10 different genes. (**F**) Four-quadrant scatter plot based on a combined analysis of MeRIP-sequencing and RNA-sequencing. (**G**) IGV plot showing m6A methylation profiles for FMOD mRNA. (**H**) The expression level of FMOD was detected by western blotting. The full-length blot is presented in Fig. S5S2H. * p < 0.05, ** p < 0.01, *** p < 0.001, compared to the corresponding group

DEGs could reflect the effects of drug therapy and were widely used to screen disease markers and therapeutic targets [33]. In this study, the RNA Sequencing was performed to screen important DEGs after combination therapy. A total of 10 DEGs, CD300ID5, MYH2, FMOD, CYP2E1, Dnase1, 9930111J2IRIK2, GALNT3, CARD11, CD2, and CILP, were screened with |exp_log2FoldChange|≥1 as the screening condition. Among them, five genes, CD300ID5, MYH2, FMOD, CYP2E1, and Dnase1 were most significantly differentially expressed between the model group and EC group (Fig. 2A-B, A1: model group, A3: EC group). FMOD, MYH2, Dnase1, and CD300ID5 were significantly decreased after combination therapy, while CYP2E1 expression levels were significantly increased. For a clearer presentation, we performed differential ploidy presentation for the above five genes, as shown in Fig. 2C, the expression levels of CD300ID5, Dnase1, FMOD, and MYH2 in the model group were FPKM = 6.12, FPKM = 5.86, FPKM = 1.34, and FPKM = 2.14, respectively. After EC treatment, the expression levels of CD300ID5, FMOD, MYH2, and CYP2E1 expression levels were FPKM = 1.28, FPKM = 1.36, FPKM = 0.14, FPKM = 0.62 and FPKM = 3.35, respectively; the decrease folds were 4.79, 4.31, 9.58, 3.46 and 7.45, respectively; CYP2E1 expression level in the model group FPKM = 0.45, and after combination treatment CYP2E1 expression level FPKM = 3.35, which increased 7.45-fold. Overall, the transcriptional level of FMOD was most significantly regulated after EC treatment. Therefore, we selected FMOD as our gene of interest.

In addition, we performed functional enrichment analysis of these 10 genes, and the significantly enriched Top10 GO terms were shown in Fig. S2, which mainly include thymic T cell selection, T cell activation, regulation of neutrophil mediated cytotoxicity and other biological processes, muscle myosin complex, membrane raft, intrinsic component of endoplasmic reticulum and other cellular components, protein self-association, polypeptide N-acetylgalactosaminyl transferase, oxidoreductase activity, acting on paired and other molecular functions. These genes were mainly enriched in the TGF-β signaling pathway, NF-kappa B signaling pathway and Cell adhesion molecules (Fig. S3) [28–30].

### Analysis of M6A modification levels

The m6A modification is the most predominant form of mRNA methylation modification [34]. Abnormal m6A modification mediated by related regulatory proteins plays an important role in lung carcinogenesis and development [35]. In this study, we performed m6A sequencing on tumor tissue samples from the model and combination treatment groups. Meanwhile, we analyzed the MeRIP-seq sequencing results of the above 10 genes CD300ID5, MYH2, FMOD, CYP2E1, Dnase1, 9930111J2IRIK2, GALNT3, CARD11, CD2, CILP with |peak_diff.log2.fc| ≥ 1 as the screening condition. The results were shown in Fig. 2D-E. There were differences in the m6A modification levels of CD300ID5, MYH2, FMOD, CYP2E1, and Dnase1 between the model and EC groups. Compared with the EC group, FMOD, MYH2, and Dnase1 had higher m6A modification levels in the model group. After Endostar combined with cisplatin treatment, the m6A modification levels of FMOD, MYH2, and Dnase1 were significantly reduced, with the most prominent reduction level of FMOD. In contrast, the m6A modification levels of CYP2E1 and CD300ID5 were relatively low in the model group, and the m6A modification levels of CYP2E1 and CD300ID5 were relatively increased after combined treatment.

### Combined analysis of MeRIP-sequencing and RNA-sequencing data

We selected |peak_diff.log2.fc|≥1 and |exp_log2FoldChange|≥1 as the screening conditions to combine the results of MeRIP-sequencing and RNA-sequencing to analyze the results. A four-quadrant scatter plot of the distribution was plotted as shown in Fig. 2F. The first quadrant indicated DEGs with up-regulated methylation and also up-regulated expression; the second quadrant indicated DEGs with up-regulated methylation and also down-regulated expression; the third quadrant indicated DEGs with down-regulated methylation and also down-regulated expression; and the fourth quadrant indicated DEGs with up-regulated methylation and down-regulated expression. We then visualized the FMOD modification levels by IGV sequencing software, and the results are shown in Fig. 2G; the methylation levels of FMOD in tumor tissues were significantly reduced after the combination treatment. In order to further confirm the effect of combined treatment on FMOD expression, we examined the expression level of FMOD in the model group, endostar group, and EC group. The results are shown in Fig. 2H; in the EC group, the expression level of FMOD was significantly reduced compared with the model group. Meanwhile, compared to control group, endostar or cisplatin treatment obviously reduced the expression level of FMOD in A549 cells (Fig. S4A). As expected, compared to the single treatment group, EC treatment further reduced FMOD level in A549 cells (Fig. S4A). Thus, we confirmed that endostar combined with cisplatin treatment could significantly reduce the level of m6A methylation modification of FMOD, which in turn caused a significant reduction in the translation level of FMOD, ultimately leading to a reduction in the expression of related anti-angiogenic factors and thus exerting anti-tumor effects.

### Regulation effect of endostar combined with cisplatin on m6A methylase METTL3

**Fig. 3:**
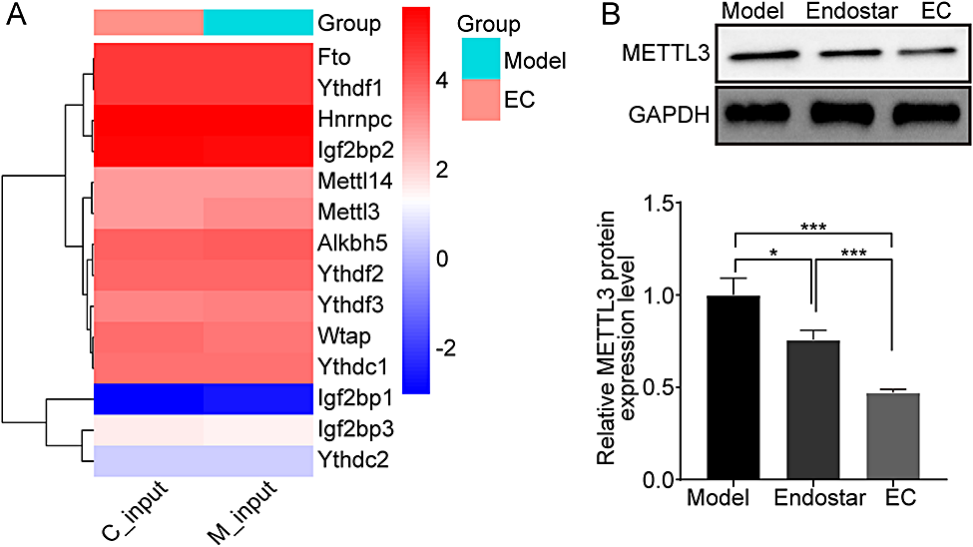
**Combination treatment down-regulates METTL3 levels in mouse tumor tissues.** (**A**) The expression heat map of several major m6A methylation-modifying enzymes. (**B**) The expression level of METTL3 was detected by western blotting. The full-length blot is presented in Fig. S5S3B

To further confirm the cause of the abnormal level of m6A methylation modification of FMOD, we extracted transcript level data of m6A methylation-modifying regulator enzymes, including METTL3, METTL14, WTAP, FTO, ALKBH5, YTHDC1, YTHDC2, YTHDF1, YTHDF2, YTHDF3, HNRNPC, IGF2BP2, IGF2BP1, and IGF2BP3. The results were shown in Fig. 3A, combination therapy could significantly inhibit the transcription level of METTL3, thus causing a decrease in the methylation level of FMOD. In addition, western blotting results further confirmed that endostar group or model group, the EC group could significantly reduce the expression level of METTL3 (Fig. 3B). Meanwhile, compared to control group, endostar or cisplatin treatment obviously declined the expression level of METTL3 in A549 cells. As expected, compared to the single treatment group, EC treatment further declined METTL3 level in A549 cells (Fig. S4B). Therefore, we speculate that combination therapy could down-regulate the transcription and translation levels of METTL3, resulting in a decrease in the methylation level of FMOD.

#### Overexpression of METTL3 could impair the anti-tumor activity of endostar combined with cisplatin

**Fig. 4:**
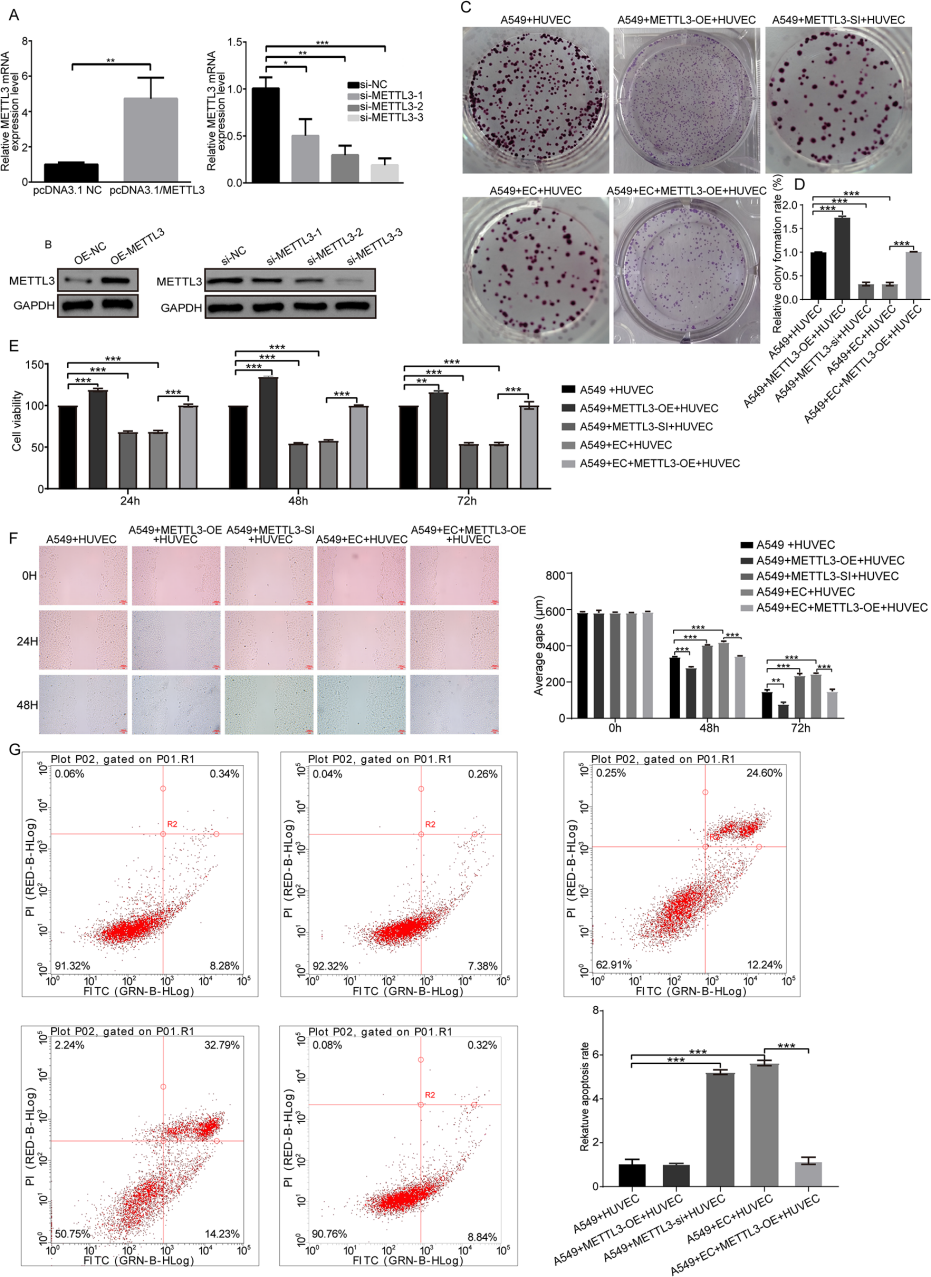
**Effect of METTL3 on anti-tumor activity of endostar combined with cisplatin.** (**A-B**) qPCR and western blotting was used to detect the expression of METTL3 after overexpression and METTL3 knockdown (siRNA silenced for 48 h) (n = 3) The full-length blot is presented in Fig. S5S4B. (**C-D**) Colony formation ability of A549 and HUVECs co-culture system under different treatment conditions. (**E**) Cell viability was measured by MTT (mean ± SD). (**F**) Wound healing assay was used to assess cell migration in each group. (**G**) Flow cytometry was used to detect cell apoptosis in each group. * p < 0.05, ** p < 0.01, *** p < 0.001, compared to the corresponding group

As mentioned above, we found that endostar combined with cisplatin could reduce the m6A methylation modification of FMOD by decreasing the expression level of METTL3. To investigate whether the expression level of METTL3 affects the therapeutic effect of combination therapy in NSCLC, we constructed METTL3 overexpression and knockdown cell lines (Fig. 4A-B). As shown in Fig. 4C-F, endostar combined with cisplatin could significantly reduce the colony formation ability, migration, and cell viability of A549 and HUVEC co-culture system. However, these pharmacological effects could be reversed by overexpression of METTL3. The results of the flow cytometry showed that overexpression of METTL3 facilitated the survival rate of A549 cells after the combination treatment (Fig. 4G).

### Overexpression of METTL3 abrogated the anti-tumor angiogenic effect of endostar combined with cisplatin

**Fig. 5:**
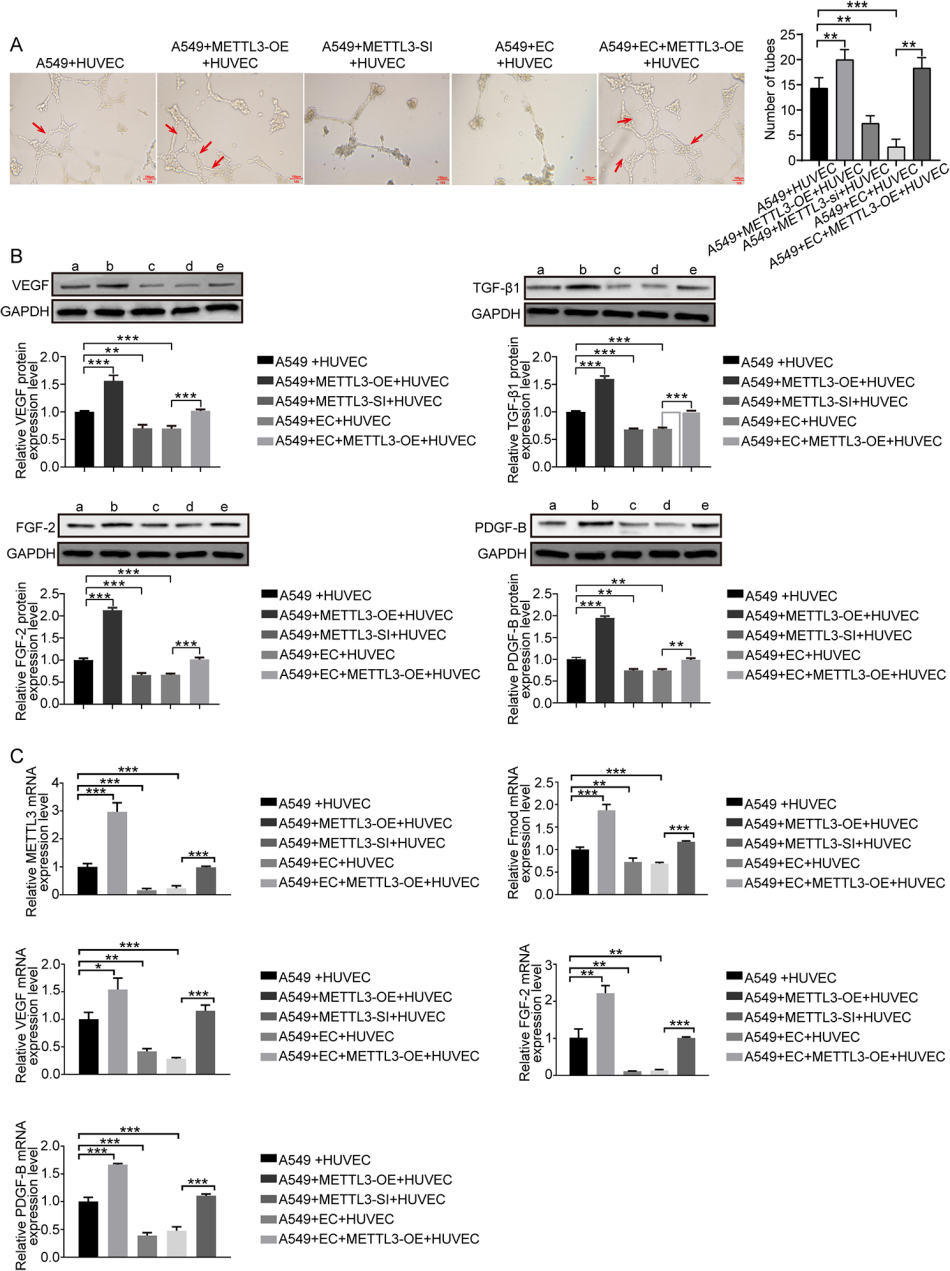
**Analysis of tumor angiogenesis.** (**A**) HUVECs were co-cultured with A549 cells with METTL3 knockdown or overexpression. (**B**) The expressions of VEGF, TGF-β1, FGF-2 and PDGF-B in different treatment groups were detected by western blotting. a: A549 + HUVEC; b: A549 + METTL3-OE + HUVEC; c: A549 + METTL3-SI + HUVEC; d: A549 + EC + HUVEC; e: A549 + EC + METTL3-OE + HUVEC. (**C**) The mRNA levels of METTL3, FMOD, VEGF, FGF-2 and PDGF-B in different treatment groups were detected by qPCR. The full-length blot is presented in Fig. S5S5B. * p < 0.05, ** p < 0.01, *** p < 0.001, compared to the corresponding group

Blood vessels in tumor tissues provide oxygen and nutrients that drive tumor growth and facilitate distant tumor metastasis [36]. HUVECs could form tubular structures in the presence of a matrix and are therefore widely used in tumor angiogenesis experiments [37]. In this study, we constructed a co-culture system of A549 and HUVECs to investigate the effect of endostar combined with cisplatin treatment on the tube formation ability of the HUVECs. As shown in Fig. 5A, we found that both combination therapy and knockdown of METTL3 expression could disrupt the tube formation ability of the co-culture system. At the same time, overexpression of METTL3 facilitated the formation of tubular lumen and abrogated the intervention effect of endostar combined with cisplatin. In addition, western blotting and qPCR results showed that overexpression of METTL3 antagonized the downregulation of VEGF, TGF-β1, FGF2, and PDGF-B expression induced by the combination treatment (Fig. 5B-C).

### Overexpression of METTL3 attenuated the effect of endostar combined with cisplatin on NSCLC mice

**Fig. 6:**
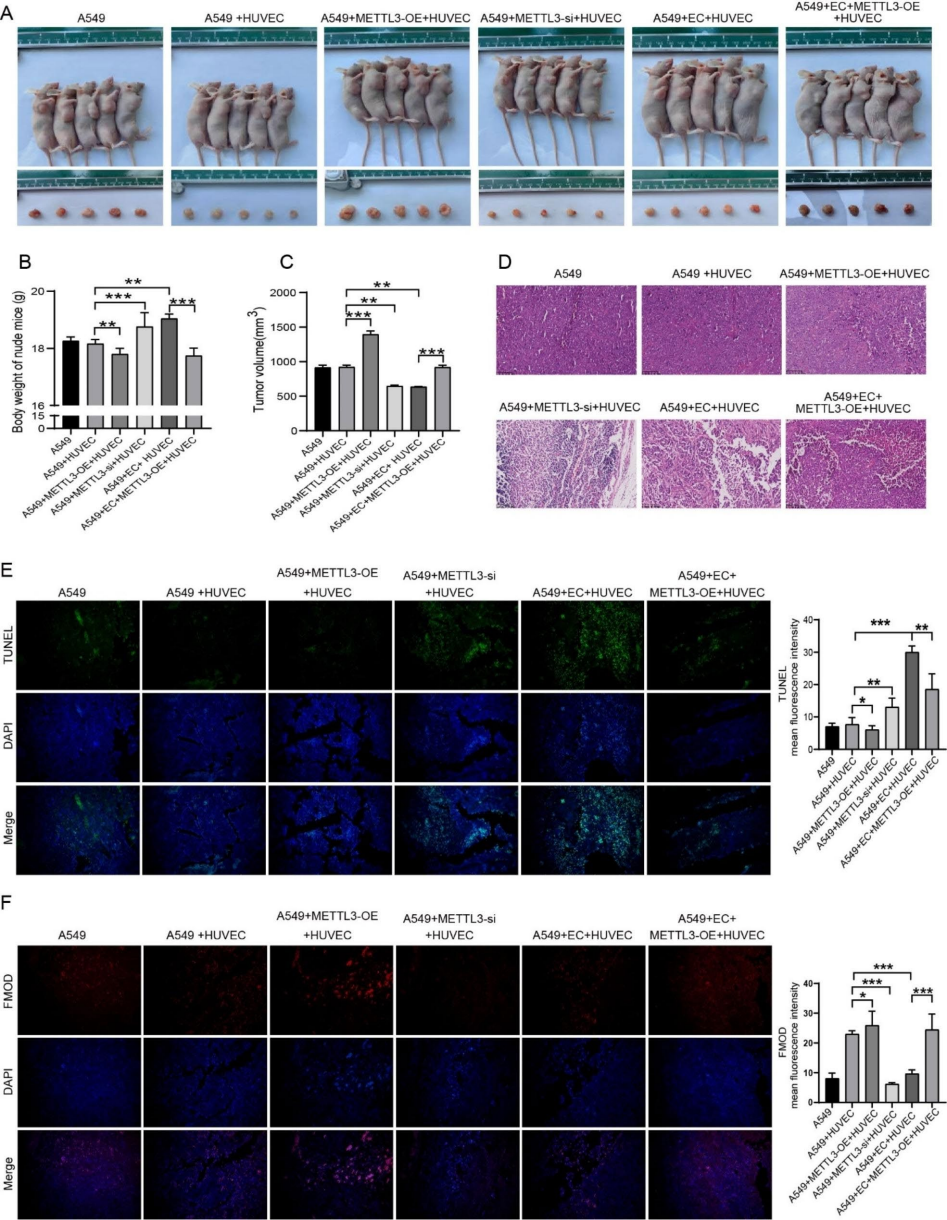
**The effect of METTL3 deficiency or overexpression on combination therapy in vivo.** (**A**) Observation of tumor formation and size of xenograft tumor tissues in each group under different treatment conditions. (**B**) Body weight of nude mice in each group (n = 5). (**C**) Tumor volume (n = 5). (**D**) HE staining was used to analyze the pathological changes of tumor tissue in each group. (**E**) TUNEL staining was used to analyze the apoptosis level of tumor cells in each group. (**F**) The expression level of FMOD was detected by immunofluorescence

**Fig. 7:**
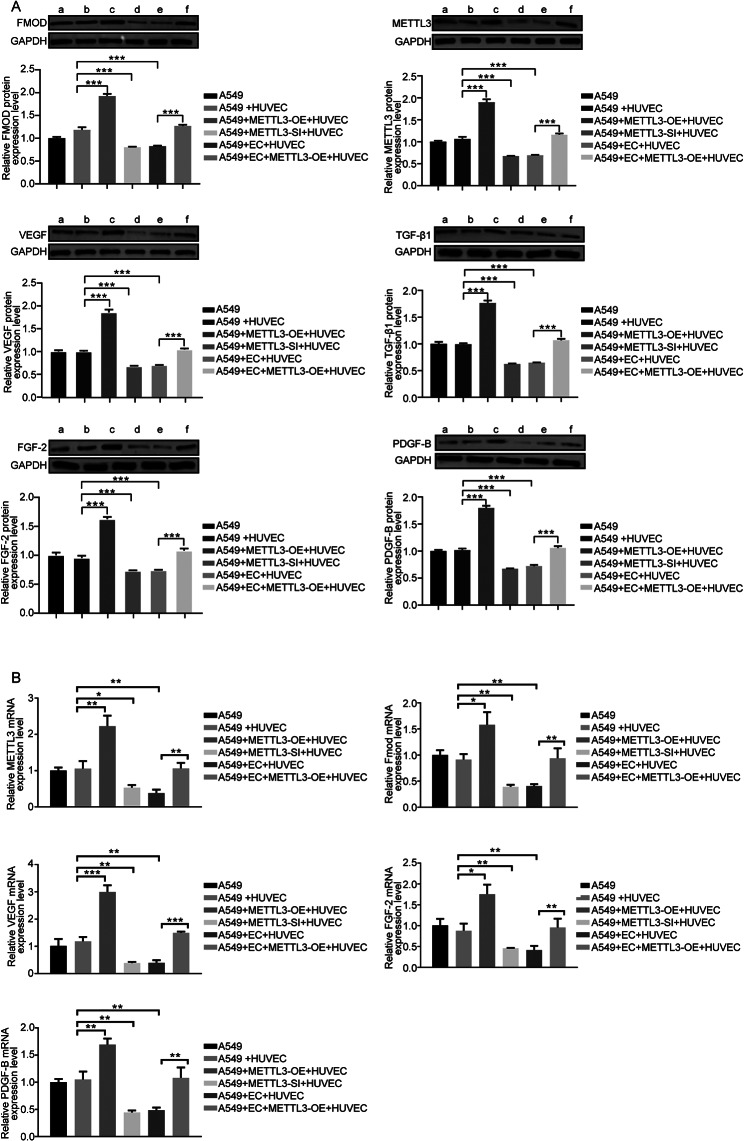
**The expression levels of METTL3 and angiogenesis indexes were quantitatively analyzed.** (**A**) The expressions levels of FMOD, METTL3, VEGF, TGF-β1, FGF-2 and PDGF-B in tumor tissues of mice in each group were detected by western blotting. a: A549; b: A549 + HUVEC; c: A549 + METTL3-OE + HUVEC; d: A549 + METTL3-SI + HUVEC; e: A549 + EC + HUVEC; f: A549 + EC + METTL3-OE + HUVEC. The full-length blot is presented in Fig. S5S7A. (**B**) The mRNA levels of FMOD, METTL3, VEGF, FGF-2 and PDGF-B in tumor tissues of mice in each group were detected by qPCR. * p < 0.05, ** p < 0.01, *** p < 0.001, compared to the corresponding group

In order to further explore the effect of METTL3 on endostar combined with cisplatin in the treatment of NSCLC mice, we constructed METTL3 overexpression and knockdown cell lines to construct a mouse tumor-bearing model. As shown in Fig. 6A-C, the tumors in the METTL3 overexpression group had the fastest growth rate, the largest size, and significant weight loss, while METTL3 interference had the opposite effect. Endostar combined with cisplatin could slow down the growth rate of tumors in vivo and increase the body weight of mice. HE and TUNEL staining showed that overexpression of METTL3 slowed down the apoptosis of cancer cells and hindered the therapeutic effect of endostar in combination with cisplatin (Fig. 6D-E). Figure 6 F showed that in HUVEC and A549 co-culture system, overexpression of METTL3 in A549 cells could improve FMOD expression level, while knockdown of METTL3 expression had the opposite effect. Combination therapy could reduce the increase of FMOD caused by METTL3 overexpression. Further, western blotting and qPCR results showed that endostar combined with cisplatin could downregulate the expression of FMOD, VEGF, TGF-β1, FGF2, and PDGF-B (Fig. 7A-B), indicating the potential anti-angiogenic effect of the combination therapy. Overexpression of METTL3 restored the expression of these proteins and might have a role in promoting the vascularization of the tumor microenvironment. The above evidence suggested that at the in vivo level, overexpression of METTL3 could attenuate the effect of combination therapy with endostar and cisplatin.

## Discussion

NSCLC is an epithelial cell-derived lung cancer with a 5-year survival rate of less than 15%, which is a serious threat to patients’ lives [38]. Despite advances in early screening, adjuvant, and neoadjuvant therapies, the average survival for advanced NSCLC patients is only 8–10 months [39, 40]. There is a clear need to rethink the treatment for NSCLC.

Angiogenesis is important in tumor development and metastasis due to the supply of oxygen and nutrients to induce tumor growth [41]. The anti-angiogenic approach is a promising strategy against tumors. Endostar is a broad-spectrum anti-angiogenic drug. Previous studies have shown that endostar blocked pathological angiogenesis to inhibit tumor formation and metastasis by binding with multiple targets [42]. It was found that endostar combined with radiotherapy or chemotherapy had excellent application prospects [22, 43]. In this study, we evaluated the therapeutic effect of endostar in combination with cisplatin in NSCLC mice. We found that the combination treatment group could significantly inhibit the colony-forming ability, migration ability and induce apoptosis of tumor cells. The combination treatment was superior to endostar alone, suggesting that endostar combined with cisplatin is an effective treatment option for NSCLC. Combination therapy has shown a powerful ability to disrupt tumor angiogenesis. FMOD is an extracellular matrix protein found mainly in connective tissues, such as skin, tendon, and cartilage. Initially, FMOD was thought to regulate ECM composition by interacting with type I collagen [44]. Recent studies have shown that FMOD acted as an angiogenic factor in tumor angiogenesis, which suggested that silencing FMOD might be a potential clinical therapy for anti-tumor [8]. The cytokines that promote tumor angiogenesis also include VEGF, FGF, PDGF, and TGF-β, etc. [45]. In this study, we found that the combination treatment significantly reduced the expression level of FMOD, and the expression of FGF-2, TGF-β1, VEGF, and PDGF-B was significantly inhibited after co-culture of FMOD-restricted A549 cells with HUVECs. It is not clear how FMOD affects the expression of these indicators, and a previous study suggested that this might be related to the involvement of FMOD in the expression and secretion of VEGF [10].

The mechanism of angiogenesis in NSCLC is still unclear, and the mechanism of endostar combined with cisplatin in the treatment of NSCLC needs to be further explored. In this study, we performed high-throughput sequencing to reveal the transcriptome and m6A landscape after combination therapy. By combining MeRIP-sequencing and RNA-sequencing data, we found five genes with differential m6A methylation modification and synchronous differential expression, including CD300ID5, MYH2, FMOD, CYP2E1, and Dnase1. Among which, the m6A modification level of FMOD was most significantly down-regulated after combination therapy. The m6A modification is a reversible process regulated by “writer”, “reader”, and “eraser” [46]. The “writer” refers to the methyltransferase complex, which catalyzes the m6A modification process. The “eraser” is a demethylase that removes m6A. RNA reader protein recognizes m6A, binds RNA, and performs the corresponding function [47]. Based on RNA-sequencing data, we found that the expression level of an m6A “writer”-METTL3 was significantly decreased after combination treatment. In addition, we demonstrated that overexpression of METTL3 could improve FMOD transcription and translation levels using qPCR and western blotting techniques, suggesting that regulating the level of METTL3 could affect the expression of FMOD.

Notably, overexpression of METTL3 almost abolished the therapeutic effect of endostar combined with cisplatin both in vivo and in vitro. Emerging evidence suggested that METTL3 played a key role in various cancers. METTL3 is highly expressed and was identified as a carcinogen in thymic epithelial tumors [48]. In gastric cancer patients, a high level of METTL3 was significantly associated with a low survival rate, and knockout of METTL3 could effectively inhibit the proliferation, migration, and invasion of gastric cancer cells [49]. Moreover, METTL3 has been shown to promote angiogenesis in gastric cancer [50]. In a mouse model of bladder cancer, METLL3 deletion inhibited tumor angiogenesis, which might be associated with the inhibition of tyrosine kinase endothelium and VEGF-A [51]. This is consistent with our findings. Briefly, METTL3 affected the stability of FMOD mRNA by regulating the m6A methylation modification of FMOD, thus affecting angiogenesis, which might interfere with the anti-tumor function of endostar combined with cisplatin. One limitation in this study is that only one lung cancer cell line was used to investigate the anti-tumor role of endostar and cisplatin. Thus, further studies are needed to validate the anti-tumor role of endostar plus cisplatin in two or more lung cancer cell lines.

## Conclusion

In conclusion, we identified the two key genes METTL3 and FMOD of endostar combined with cisplatin in the treatment of NSCLC through RNA sequencing and m6A sequencing. Briefly, METTL3 acted as the m6A methylation modification “writer” of FMOD, which stabilized FMOD mRNA and contributed to the pro-angiogenic effect of FMOD. Endostar combined with cisplatin might exert anti-tumor effects by down-regulating the expression of METTL3 and FMOD. This work elucidated the pharmacological effects and molecular mechanisms of endostar combined with cisplatin against NSCLC and further supported that METTL3 and FMOD could serve as therapeutic targets for NSCLC.

### Electronic supplementary material

Below is the link to the electronic supplementary material.

Supplementary Material 1

## Data Availability

All data generated or analyzed during this study are included in this published article.
