# Supplementary material for: Multi-omics sequencing revealed endostar combined with cisplatin treated non small cell lung cancer via anti-angiogenesis
Source: BMC Cancer. 2024 Feb 8;24:187. doi: 10.1186/s12885-023-11665-w (PMC10854066; doi:10.1186/s12885-023-11665-w)
Supplement: Supplementary file 1 — Supplementary Material 1 [file 12885_2023_11665_MOESM1_ESM.pdf]

Figure S1 Anti-tumor effect of endostar combined with cisplatin in vitro. A549 cells were treated with endostar, cisplatin or endostar + cisplatin. (A) Cell viability was measured by MTT. (B) Cell proliferation was determined by colony formation assay. (C) Wound healing assay was used to assess cell migration. (D) Flow cytometry was used to detect cell apoptosis. * $p < 0.05$, ** $p < 0.01$, *** $p < 0.001$, compared to the corresponding group.

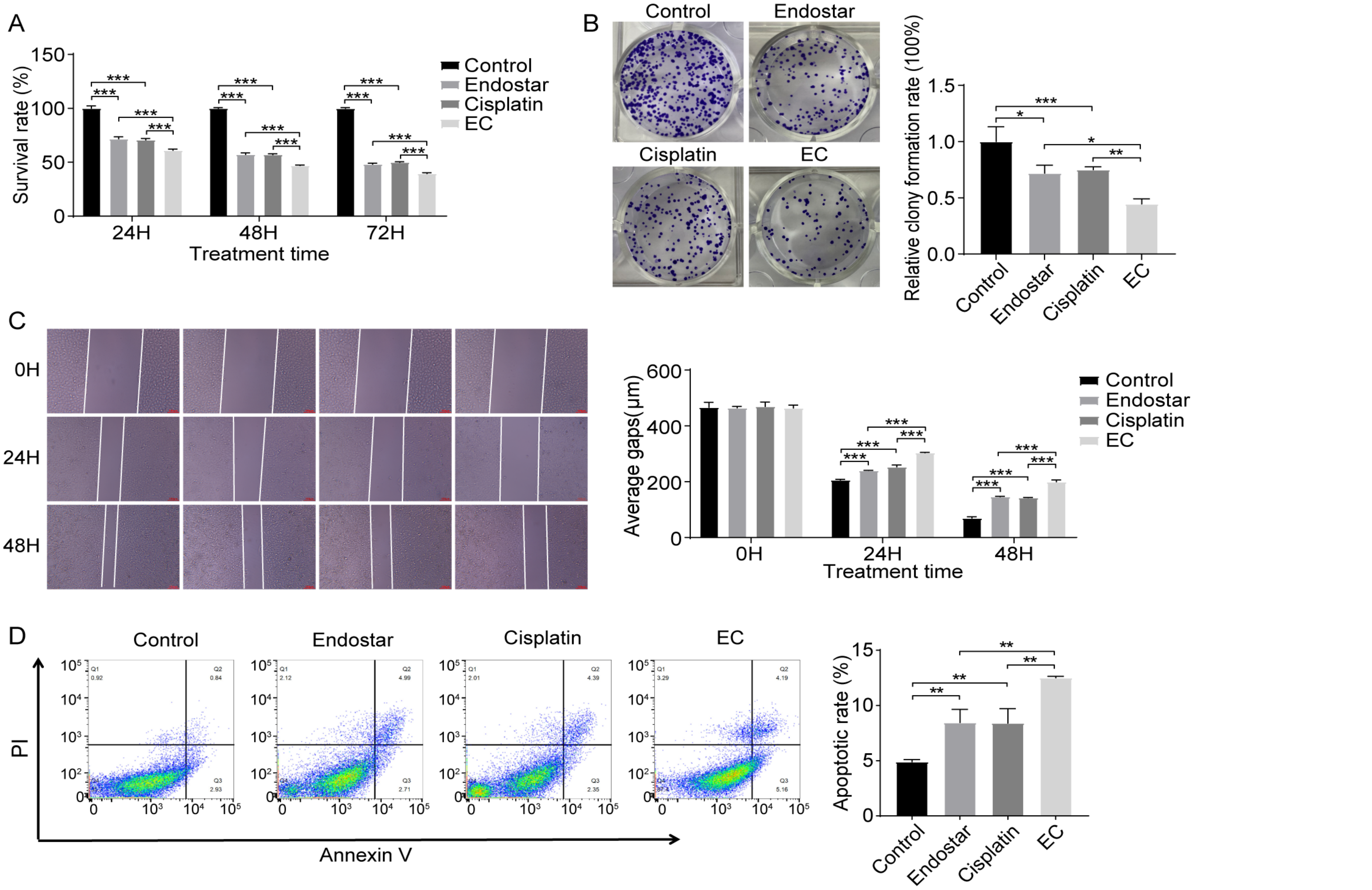

Figure S2. Top10 GO terms

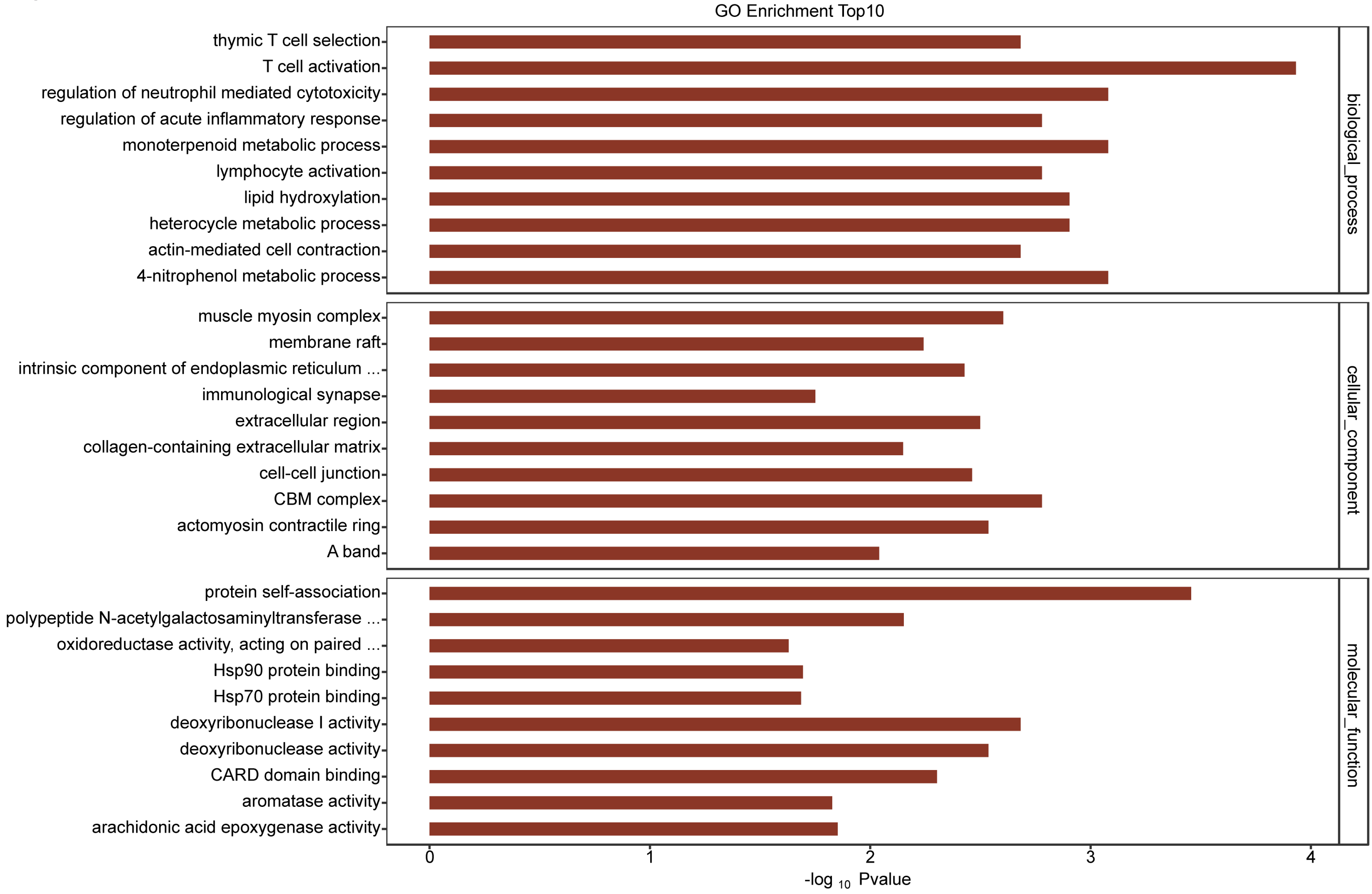

Figure S3. KEGG pathway enrichment analysis

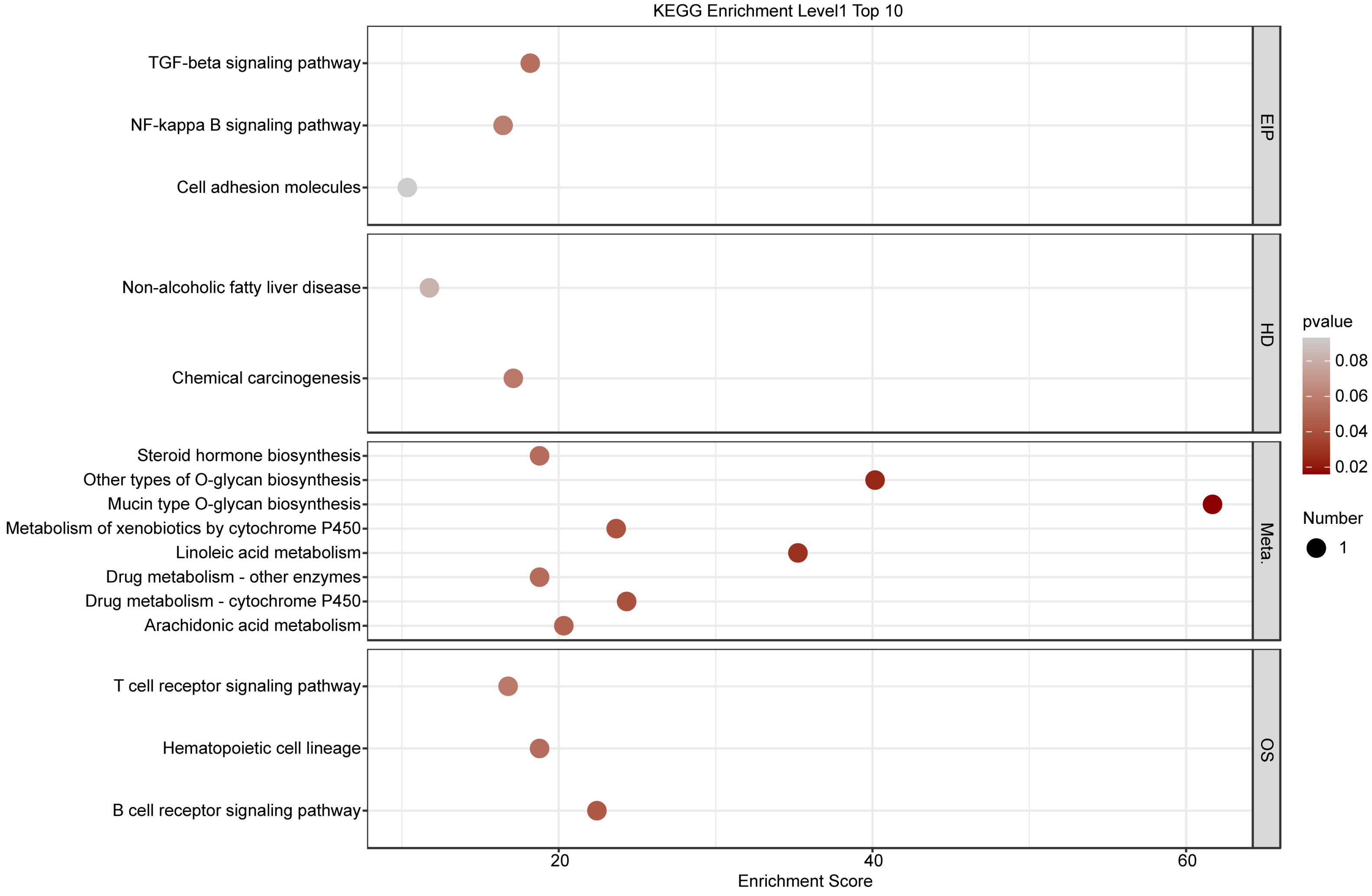

Figure S4 FMOD and METTL3 expression levels in A549 cell treated with endostar combined with cisplatin in vitro. A549 cells were treated with endostar, cisplatin or endostar + cisplatin. (A, B) The expression levels of FMOD and METTL3 in A549 cells were detected by western blotting. * $p < 0.05$, ** $p < 0.01$, *** $p < 0.001$, compared to the corresponding group.

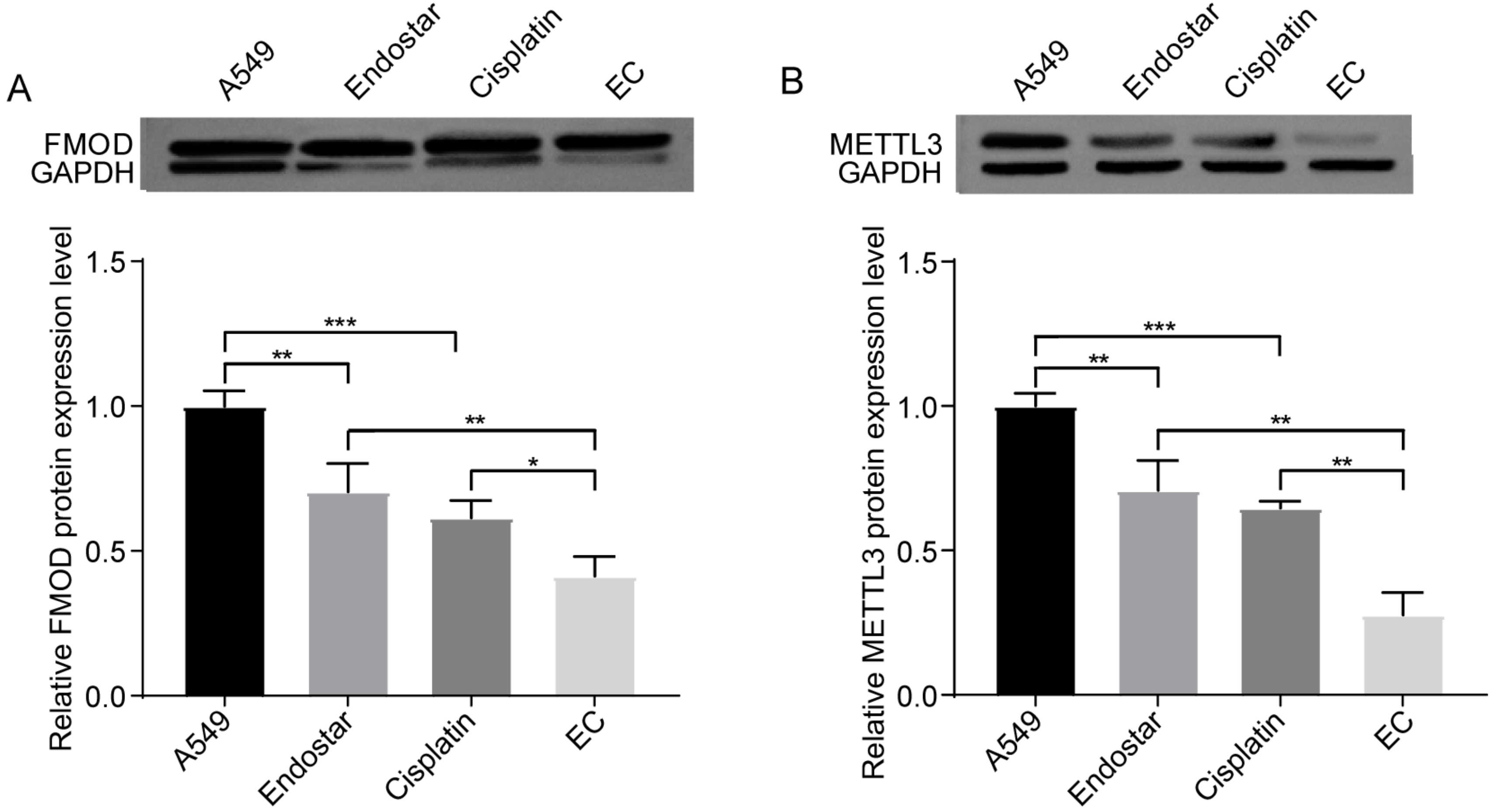

Figure S5 Full-length blot for Western blotting image-v3

S2H

FMOD

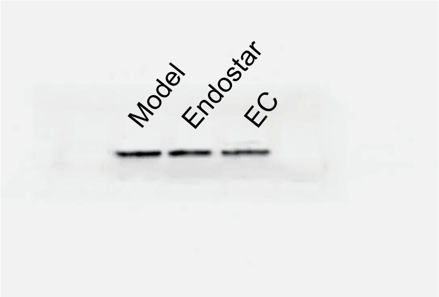

GAPDH

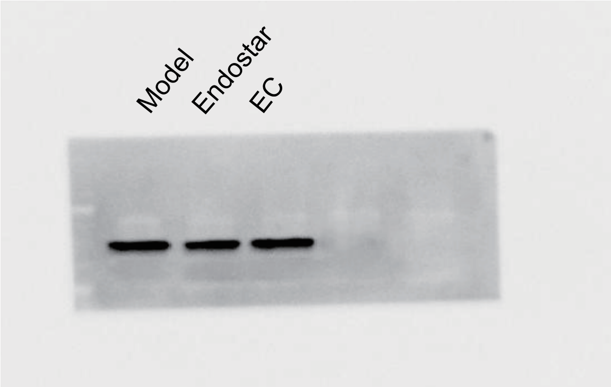

S3B

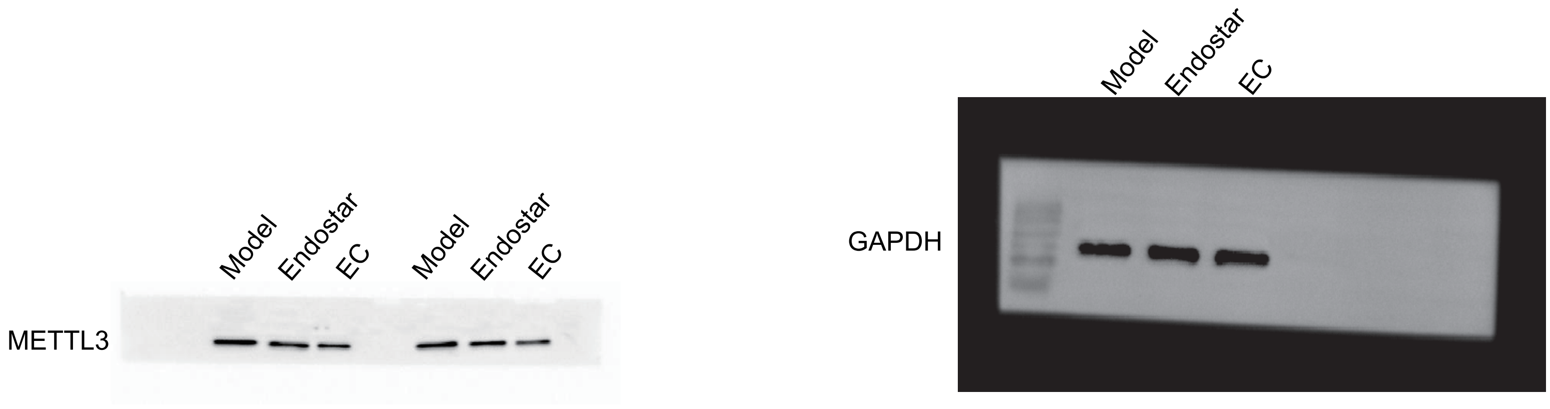
Model
Endostar
EC
Model
Endostar
EC
METTL3
Model
Endostar
EC
GAPDH

S4B

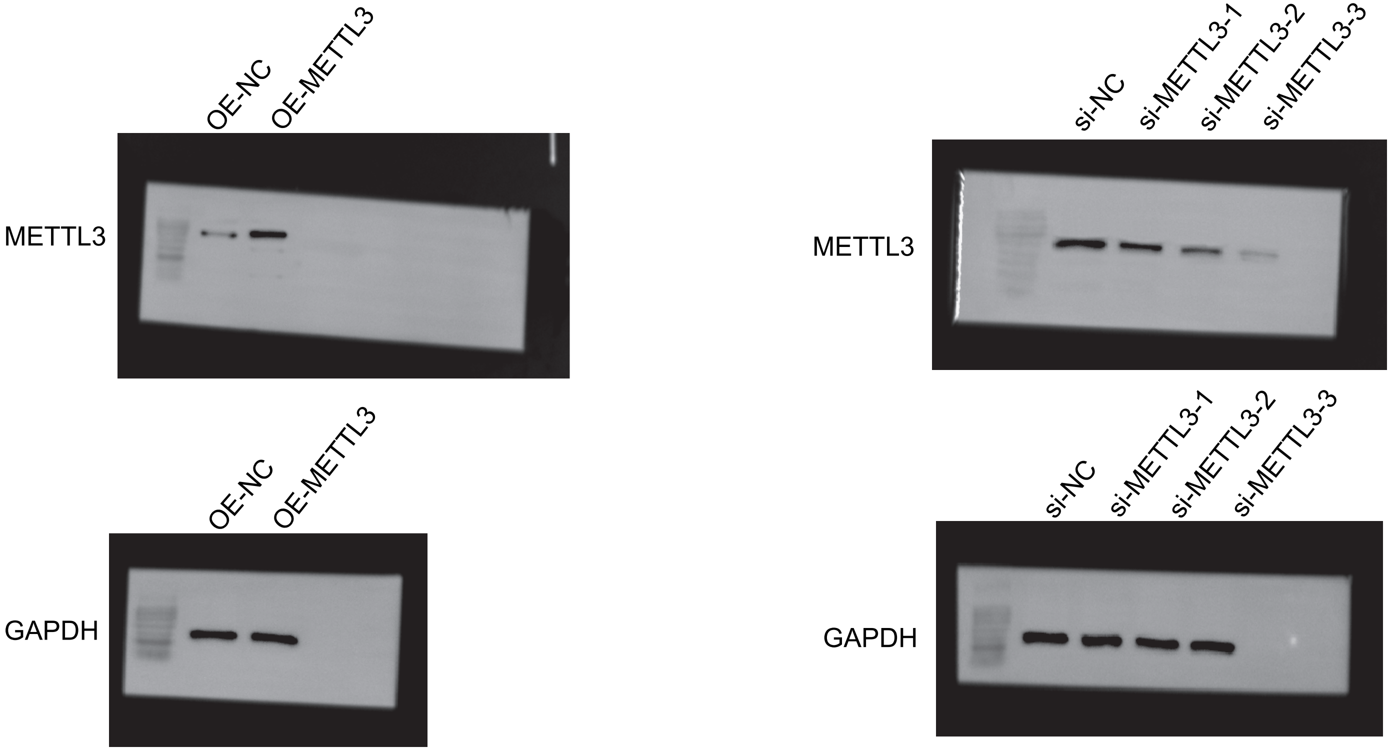
OE-NC
OE-METTL3
METTL3
si-NC
si-METTL3-1
si-METTL3-2
si-METTL3-3
METTL3
OE-NC
OE-METTL3
GAPDH
si-NC
si-METTL3-1
si-METTL3-2
si-METTL3-3
GAPDH

S5B

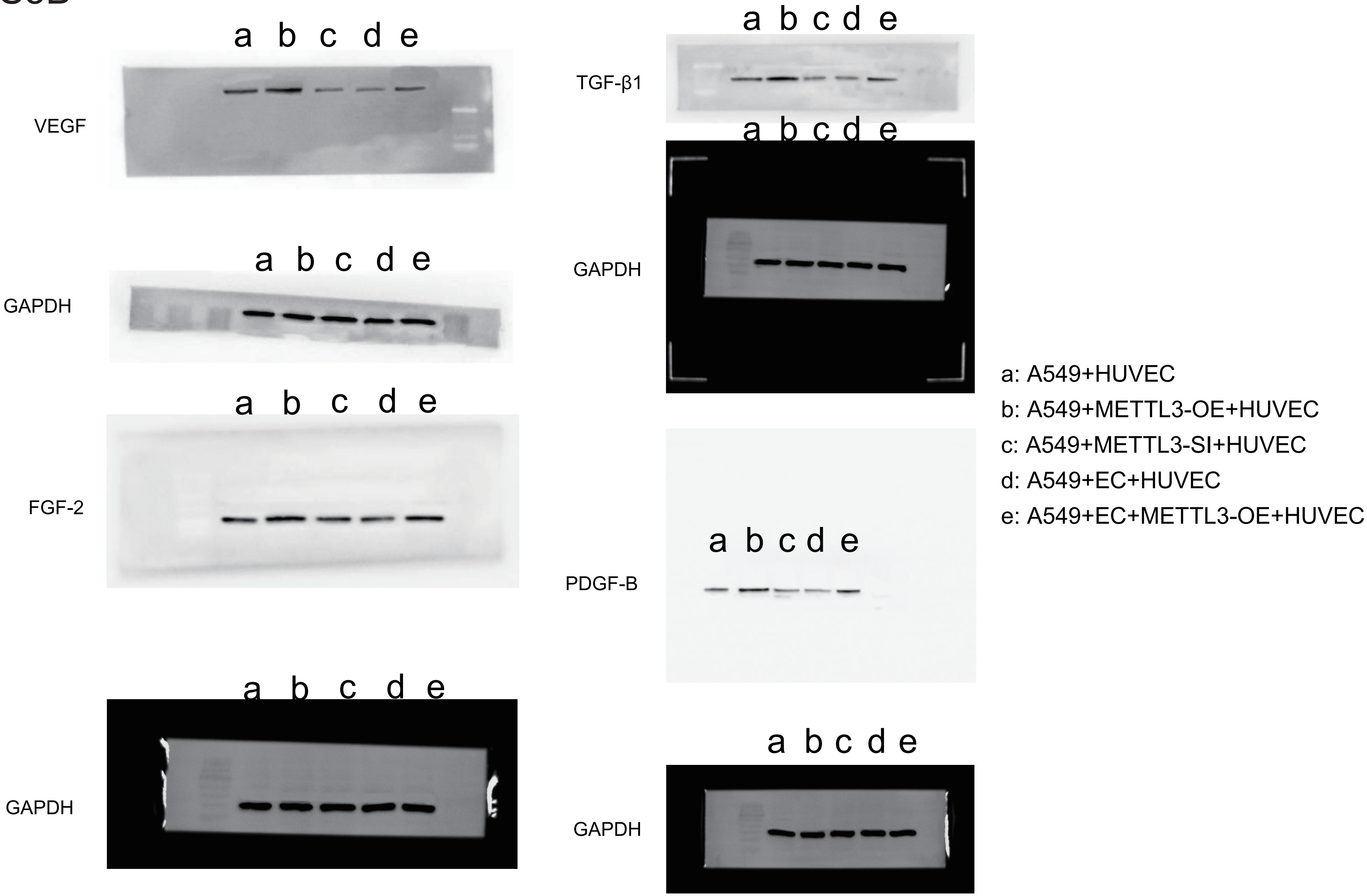
a b c d e
VEGF
GAPDH
FGF-2
GAPDH
TGF-β1
GAPDH
PDGF-B
GAPDH
a: A549+HUVEC
b: A549+METTL3-OE+HUVEC
c: A549+METTL3-SI+HUVEC
d: A549+EC+HUVEC
e: A549+EC+METTL3-OE+HUVEC

S7A

a: A549
b: A549+HUVEC
c: A549+METTL3-OE+HUVEC
d: A549+METTL3-SI+HUVEC
e: A549+EC+HUVEC
f: A549+EC+METTL3-OE+HUVEC

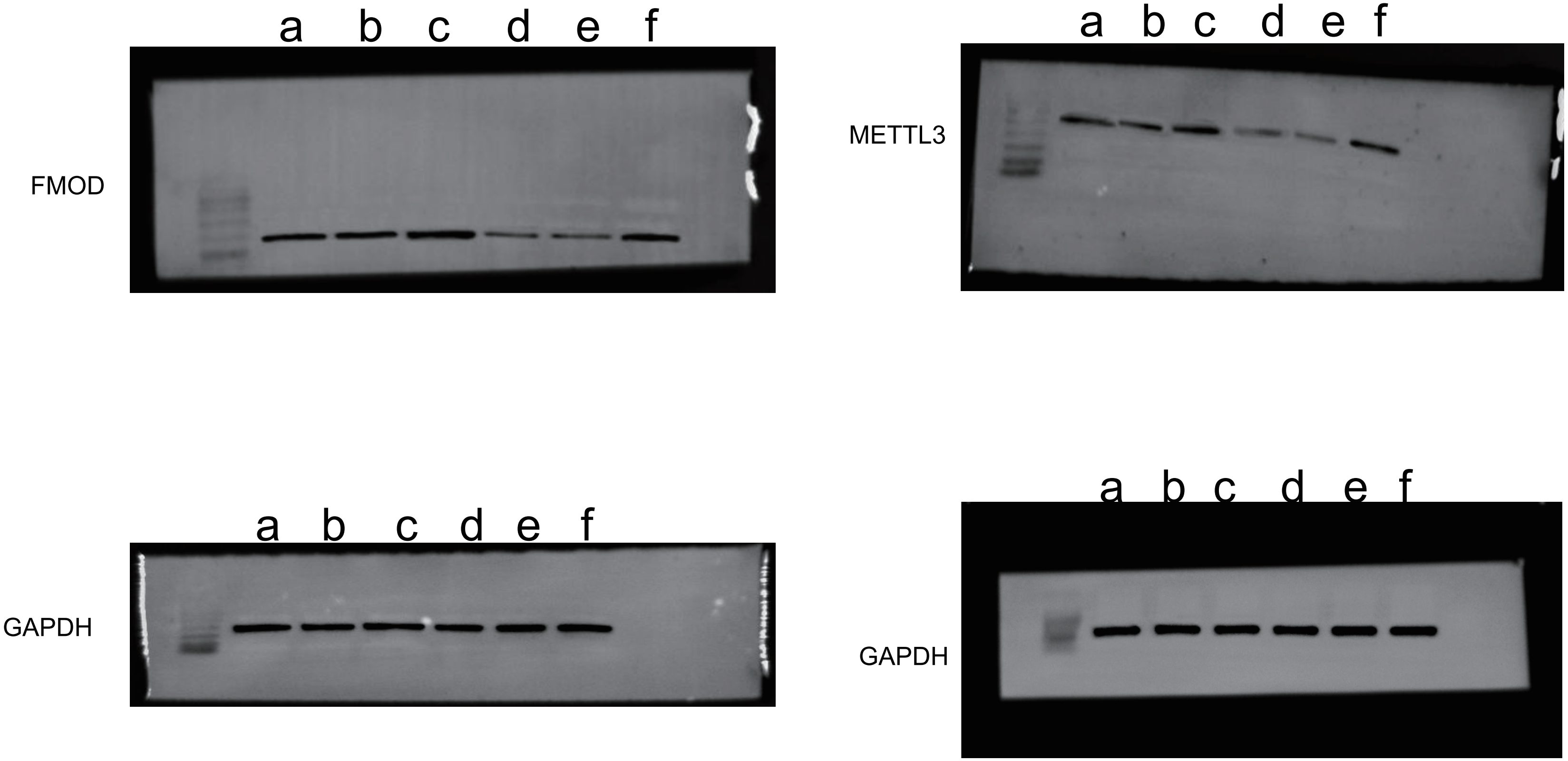

S7A

a: A549
b: A549+HUVEC
c: A549+METTL3-OE+HUVEC
d: A549+METTL3-SI+HUVEC
e: A549+EC+HUVEC
f: A549+EC+METTL3-OE+HUVEC

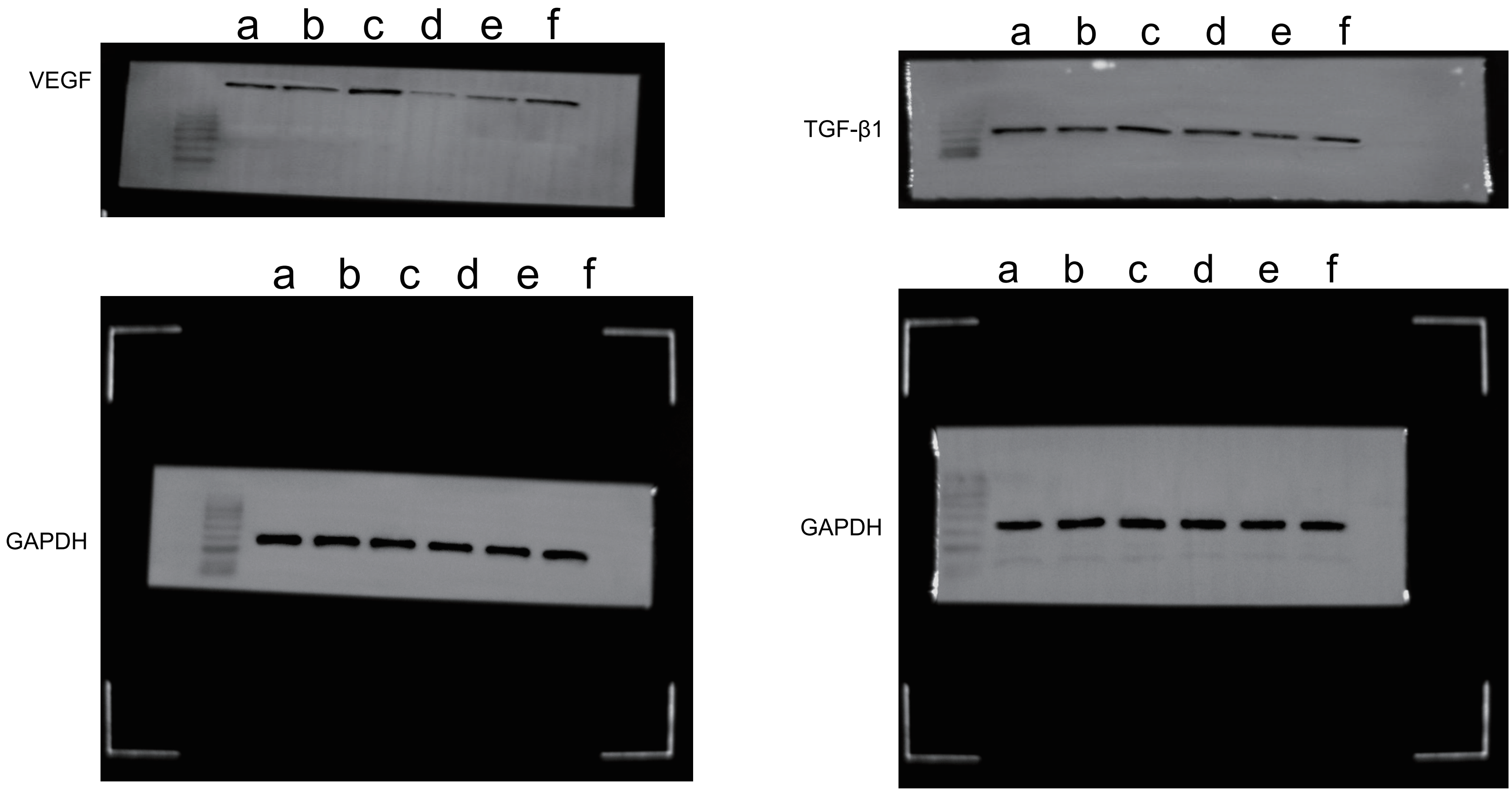

S7A

a: A549
b: A549+HUVEC
c: A549+METTL3-OE+HUVEC
d: A549+METTL3-SI+HUVEC
e: A549+EC+HUVEC
f: A549+EC+METTL3-OE+HUVEC

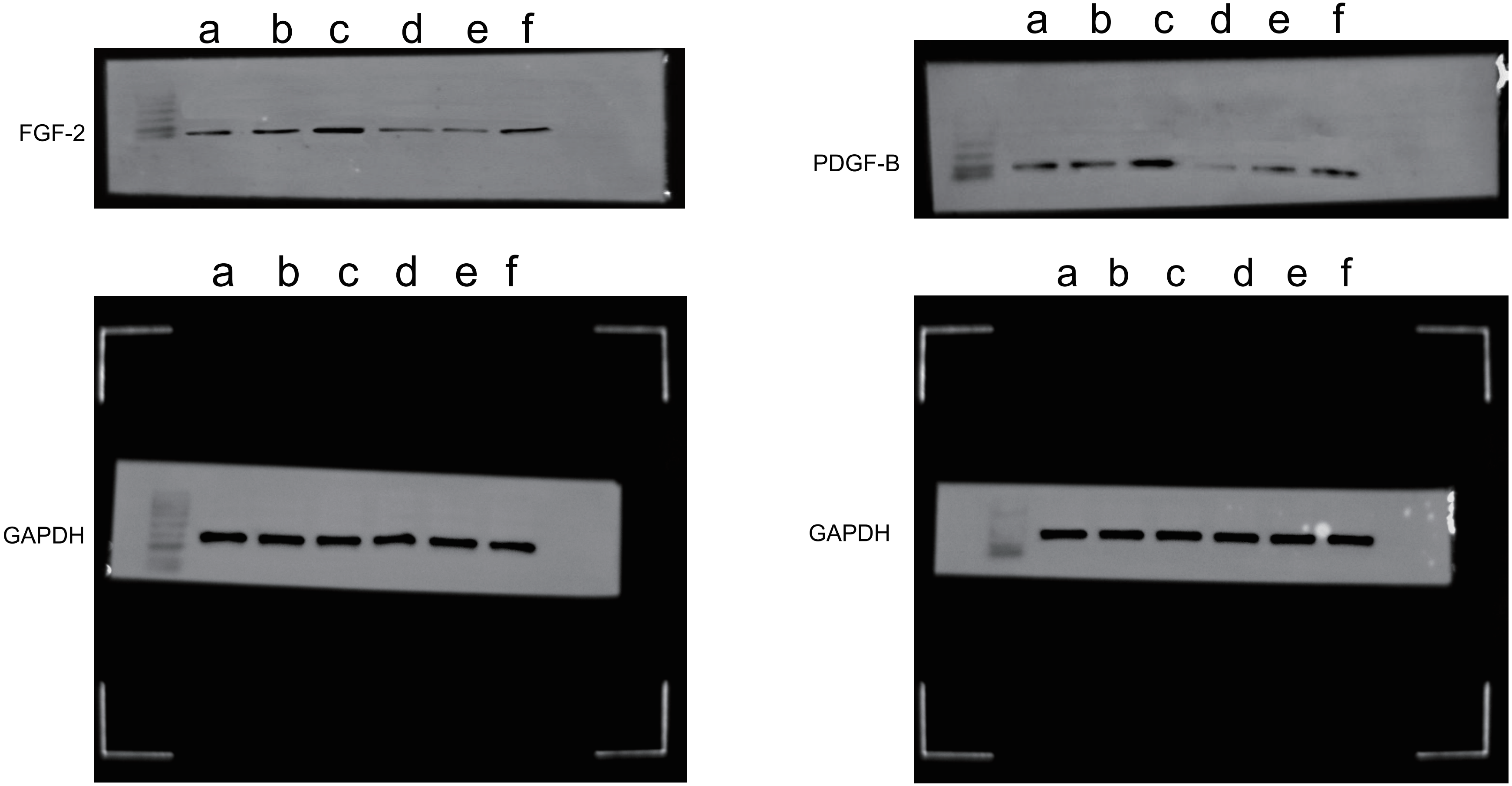

Table S1. The results of MTT

MTT							
24H		Calibration well	A549+HUVEC	A549+METTL3-OE+HUVEC	A549+METTL3-si+HUVEC	A549+EC+HUVEC	A549+EC+METTL3-OE+HUVEC
		0.037	0.618	0.719	0.427	0.431	0.612
		0.034	0.613	0.734	0.436	0.441	0.624
		0.041	0.62	0.723	0.433	0.429	0.616
	Calibrated data	Calibration well	A549+HUVEC	A549+METTL3-OE+HUVEC	A549+METTL3-si+HUVEC	A549+EC+HUVEC	A549+EC+METTL3-OE+HUVEC
		0	0.581	0.682	0.39	0.394	0.575
		0	0.579	0.7	0.402	0.407	0.59
		0	0.579	0.682	0.392	0.388	0.575
	Cell viability		100.00	117.38	67.13	67.81	98.97
			100.00	120.90	69.43	70.29	101.90
			100.00	117.79	67.70	67.01	99.31
	Mean		100.00	118.69	68.09	68.37	100.06
	SD		0.00	1.92	1.20	1.71	1.60
48H		Calibration well	A549+HUVEC	A549+METTL3-OE+HUVEC	A549+METTL3-si+HUVEC	A549+EC+HUVEC	A549+EC+METTL3-OE+HUVEC
		0.042	1.082	1.438	0.598	0.629	1.081
		0.039	1.081	1.449	0.614	0.64	1.086
		0.043	1.091	1.441	0.611	0.658	1.078
	Calibrated data	Calibration well	A549+HUVEC	A549+METTL3-OE+HUVEC	A549+METTL3-si+HUVEC	A549+EC+HUVEC	A549+EC+METTL3-OE+HUVEC
		0	1.04	1.396	0.556	0.587	1.039
		0	1.042	1.41	0.575	0.601	1.047
		0	1.048	1.398	0.568	0.615	1.035
	Cell viability		100.00	134.23	53.46	56.44	99.90
			100.00	135.32	55.18	57.68	100.48
			100.00	133.40	54.20	58.68	98.76
	Mean		100.00	134.31	54.28	57.60	99.71
	SD		0.00	0.96	0.86	1.12	0.88
72H		Calibration well	A549+HUVEC	A549+METTL3-OE+HUVEC	A549+METTL3-si+HUVEC	A549+EC+HUVEC	A549+EC+METTL3-OE+HUVEC
		0.038	1.609	1.839	0.858	0.851	1.532
		0.040	1.520	1.759	0.855	0.856	1.562
		0.039	1.568	1.836	0.868	0.871	1.606
	Calibrated data	Calibration well	A549+HUVEC	A549+METTL3-OE+HUVEC	A549+METTL3-si+HUVEC	A549+EC+HUVEC	A549+EC+METTL3-OE+HUVEC
		0	1.571	1.801	0.82	0.813	1.494
		0	1.48	1.719	0.815	0.816	1.522
		0	1.529	1.797	0.829	0.832	1.567
	Cell viability		100.00	114.64	52.20	51.75	95.10
			100.00	116.15	55.07	55.14	102.84
			100.00	117.53	54.22	54.41	102.49
	Mean		100.00	116.11	53.83	53.77	100.14
	SD		0.00	1.44	1.48	1.78	4.37
